# Navigating the Intersection of Lasers and the Skin Microbiome: A New Frontier in Esthetic Dermatology

**DOI:** 10.1111/jocd.70150

**Published:** 2025-04-01

**Authors:** Diala Haykal, Marco Rocha

**Affiliations:** ^1^ Centre Laser Palaiseau, Private Practice Palaiseau France; ^2^ Federal University of São Paulo São Paulo Brazil

**Keywords:** cosmetic dermatology, microbiology, non‐ablative lasers

## 1 Introduction

Advances in esthetic dermatology have increasingly emphasized the role of laser technologies in achieving precise, non‐invasive, and highly effective treatments for various skin concerns. Lasers have become a cornerstone of modern dermatological care, offering solutions for conditions such as acne scars, hyperpigmentation, and skin laxity [1]. While the immediate therapeutic benefits of laser treatments are well‐documented, a significant gap remains in understanding their broader systemic effects, particularly on the skin microbiome [2]. This oversight is critical because the skin microbiome functions as an integral component of skin health, influencing everything from immune defense to wound healing [3, 4]. By intentionally disrupting the skin's structure to stimulate repair and rejuvenation, laser treatments may inadvertently affect this vital ecosystem [5, 6]. Despite the increasing integration of microbiome research into dermatology, there remains limited direct clinical evidence demonstrating long‐term microbiome disruption following laser procedures. While some hypotheses suggest that ablative lasers could alter microbial populations, no large‐scale studies confirm an increased risk of infection or impaired healing due to microbiome dysbiosis. Instead, the skin's regenerative mechanisms often restore its microbial equilibrium post‐treatment. Nevertheless, understanding how different laser modalities interact with the skin microbiome is an area requiring further investigation to optimize treatment protocols and improve patient outcomes. The question of how to balance therapeutic goals with microbiome preservation is now at the forefront of esthetic medicine. The skin microbiome plays a pivotal role in maintaining immune defense, skin barrier function, and overall dermatological health [7, 8, 9]. Recent studies underscore its vulnerability to various interventions, including laser treatments, highlighting the need for microbiome‐conscious strategies. This commentary aims to bridge the gap between laser advancements and their impact on the microbiome, underscoring the importance of integrating microbiome‐friendly practices into dermatology.

## 2 The Role of the Skin Microbiome in Skin Health

The skin microbiome operates as a dynamic and adaptive interface between the body and its external environment [10]. Its primary function is to protect the skin from pathogenic invasion by outcompeting harmful microbes and producing antimicrobial peptides. Beyond this, the microbiome also modulates local immune responses, preventing unnecessary inflammation that could damage skin tissues [11]. For example, commensal bacteria like *Staphylococcus epidermidis* and Cutibacterium acnes play critical roles in maintaining skin equilibrium. Cutibacterium acnes also contributes to maintaining the skin's acidic pH by metabolizing sebum triglycerides into short‐chain fatty acids, such as propionic acid, which help sustain the acid mantle [12]. This acidic environment is crucial for skin health, as it inhibits pathogen colonization and supports the function of pH‐dependent enzymes involved in skin barrier maintenance (Table 1). However, the fragility of this ecosystem is a concern. These organisms can be adversely affected by ablative lasers, necessitating strategies to protect or restore their populations. Incorporating topical probiotics or postbiotics post‐procedure can help reinstate microbial balance [13]. Even subtle shifts in microbial composition can lead to dysbiosis, resulting in increased susceptibility to conditions such as atopic dermatitis or rosacea [14]. Environmental stressors, such as pollution, and therapeutic interventions, like lasers, can exacerbate this imbalance. Understanding the microbiome's resilience and thresholds for disruption is essential for developing treatments that respect and preserve this equilibrium [5, 15, 16].

**TABLE 1:** Probiotic strains and their potential applications in dermatology.

Skin disorder	Probiotic strain	Proposed mechanism	Clinical insights
Acne	*Lactobacillus acidophilus*	Reduces inflammation and inhibits *C. acnes*.	Clinical trials demonstrate reduced lesion counts and improved skin texture with topical and oral use.
	*Bifidobacterium bifidum*	Balances sebum production and reduces oxidative stress.	Supplementation linked to decreased oil production in acne‐prone skin.
Atopic dermatitis	*Lactobacillus rhamnosus*	Balances Th1/Th2 immune response and reduces IgE levels.	Pediatric studies show reduced AD severity with oral supplementation.
	*Bifidobacterium lactis*	Enhances skin hydration and reduces inflammation.	Found to improve skin barrier recovery after disruption.
Rosacea	*Bifidobacterium bifidum*	Restores barrier function and reduces erythema.	Patients report reduced redness and flare frequency after regular use.
	*Lactobacillus reuteri*	Modulates the inflammatory response to triggers.	Topical formulations show promise in reducing sensitivity in rosacea patients.
Photoaging	*Lactobacillus casei*	Provides antioxidant effects and mitigates UV‐induced damage.	Clinical data support improved skin elasticity and reduced fine lines.
	*Streptococcus thermophilus*	Boosts ceramide production, enhancing skin hydration.	Associated with improved moisture retention and reduced signs of aging.
Psoriasis	*Bifidobacterium infantis*	Regulates systemic inflammation by modulating gut microbiota.	Oral supplementation reduces Psoriasis Area and Severity Index (PASI) scores in mild cases.
Eczema	*Lactobacillus paracasei*	Restores microbial balance and improves skin barrier integrity.	Studies highlight reduced itching and improved quality of life.
Sensitive skin	*Lactobacillus plantarum*	Enhances barrier repair and reduces trans‐epidermal water loss.	Associated with decreased sensitivity and redness in clinical settings.
Post‐procedure recovery	*Staphylococcus epidermidis*	Promotes wound healing and protects against opportunistic pathogens.	Shown to reduce recovery time and minimize risk of post‐laser infections.
Pigmentation disorders	*Lactobacillus fermentum*	Reduces oxidative stress and inhibits melanogenesis.	Early studies suggest reduced hyperpigmentation when used alongside topical antioxidants.

## 3 The Mechanisms of Laser Technologies and Their Effects on Skin

Laser technologies achieve their effects by delivering energy to targeted chromophores, causing controlled thermal or photomechanical damage. This damage is intended to stimulate processes like collagen remodeling, cellular turnover, or vascular constriction, depending on the laser type and clinical indication [1]. For example, CO_2_ lasers ablate the skin's surface, leading to dramatic improvements in texture and tone, but also significantly alter the physical and microbial landscape [17]. Non‐ablative lasers, on the other hand, induce sub‐epidermal changes without disrupting the skin barrier, making them less likely to perturb microbial populations [18]. Fractional lasers strike a balance by creating microcolumns of injury surrounded by intact tissue, which supports faster recovery but may still affect the local microbiota [19]. The microbial impact of these technologies is largely understudied. Ablative treatments, which remove the protective outer layer of the skin, may expose deeper tissues to opportunistic pathogens, increasing the risk of infection [20]. This disruption creates a temporary void, which opportunistic pathogens like *Staphylococcus aureus* or *Pseudomonas aeruginosa* might exploit [20, 21]. Beyond bacterial populations, the impact of lasers on the skin microbiome may extend to interactions with physiological skin phages. Potential interactions between skin phages and external factors, such as temperature changes induced by laser treatments, are emerging as an area of growing interest [22]. While much focus has traditionally been placed on bacteria within the skin microbiome, the implications of laser treatments on phages and the broader microbiome may be even more significant. Physiological skin phages, particularly those targeting Cutibacterium acnes, play a crucial role in maintaining microbial homeostasis on the skin. These phages help regulate bacterial populations, preventing overgrowth and reducing the risk of inflammation associated with conditions like acne. Dysbiosis of these phages, whether due to an imbalance in their abundance, loss of diversity, or genetic changes, can disrupt this equilibrium, potentially leading to pathogenic bacterial overgrowth and exacerbating inflammatory skin disorders [23].

By influencing local skin temperature, lasers could impact the activity, replication, and host interactions of bacteriophages. This disruption could affect microbial homeostasis, influencing not only bacterial populations, but also the immune response and skin barrier function. Furthermore, environmental factors such as UV radiation, temperature, and humidity fluctuations are known to influence phage‐bacteria interactions, highlighting the importance of exploring how laser‐induced temperature changes may impact phage dynamics. Investigating these interactions is crucial to fully understanding the impact of dermatological treatments on the microbiome, ultimately helping to optimize both the safety and efficacy of these procedures [22, 23].

Non‐ablative and fractional modalities, though gentler, could still disrupt microbial habitats, particularly with repeated or high‐intensity use. Additionally, the inflammatory response triggered by laser treatments could exacerbate microbial imbalances, especially in patients with pre‐existing conditions like rosacea, where inflammation and dysbiosis are already intertwined [24]. By altering skin hydration levels, these treatments may shift microbial communities, favoring certain species over others [25]. For instance, warm and moist conditions post‐treatment could encourage the overgrowth of yeast‐like fungi, leading to conditions such as pityrosporum folliculitis [26, 27]. This disruption in the skin's barrier and microbial balance can also facilitate the reactivation of herpes simplex virus (HSV) in individuals with a history of the virus. The reactivation of HSV after fractional laser treatment is primarily attributed to the skin's response to thermal and mechanical stress induced by the procedure. Fractional lasers create controlled microthermal zones of injury, which stimulate wound healing and collagen remodeling. However, this disruption of the skin barrier, combined with local inflammation and dysbiosis, could trigger HSV reactivation [28]. Stress‐induced immunomodulation, including temporary suppression of local immune defenses, further facilitates viral replication and reactivation. These potential consequences necessitate a deeper understanding of how laser technologies interact with the microbiome at both a cellular and ecological level.

Different types of dermatological treatments have unique effects on the skin and its microbiome, requiring careful consideration of their mechanisms. When comparing non‐laser treatments to lasers, the mechanisms of action and their effects on the skin differ significantly. While lasers target the dermis through controlled thermal injury to stimulate repair and remodeling, non‐laser treatments such as chemical peels and injectables primarily affect the skin's surface or localized areas. Chemical peels disrupt the stratum corneum, leading to exfoliation that may temporarily alter microbial diversity [29]. Injectable treatments, on the other hand, can cause localized inflammation, which may indirectly influence the microbiome by altering the skin's microenvironment. Recognizing these differences is crucial for designing treatment protocols that minimize unintended microbial disruption while optimizing therapeutic outcomes [30]. Future individualized protocols, accounting for patient history and skin microbiome conditions, could help prevent such outcomes, even without the use of antiviral medication. While current evidence highlights the potential effects of laser treatments on the skin microbiome, much of this discussion remains largely theoretical due to a lack of long‐term, controlled studies.

## 4 The Role of Combined Technologies in Minimizing Microbial Disruption

Emerging combined technologies that combine lasers with other modalities, such as photobiomodulation, present an exciting avenue for reducing the impact of treatments on the microbiome [31, 32, 33]. These combined approaches may allow for lower energy levels while still achieving comparable esthetic results, thereby minimizing the risk of disrupting microbial communities. For example, pairing fractional laser treatments with LED therapy can stimulate collagen production while promoting a favorable microbial environment [34]. While preliminary studies suggest that these combined approaches may help maintain microbial balance, there is currently insufficient clinical evidence to confirm their effectiveness in preventing microbiome disruption. Further investigations, including randomized controlled trials, are needed to validate their long‐term impact and establish standardized treatment protocols that incorporate microbiome‐friendly techniques.

## 5 The Need for Microbiome‐Friendly Approaches

Incorporating microbiome‐conscious strategies into laser protocols is both a scientific necessity and an ethical imperative. Advances in laser technology have already made it possible to achieve significant esthetic results with reduced invasiveness, but further refinements are needed to minimize collateral damage to the microbiome [10]. One promising approach is the use of non‐ablative lasers that deliver sufficient energy to stimulate dermal remodeling without compromising the epidermal barrier. Fractional technologies, particularly those with adjustable parameters, offer another avenue for balancing efficacy with microbiome preservation. Equally important is the timing and frequency of treatments. Overly aggressive protocols can overwhelm the skin's natural repair mechanisms, prolonging dysbiosis and delaying recovery. Spacing sessions appropriately allows the skin's microbial and structural systems to recover fully, reducing the risk of cumulative damage. By tailoring treatments to individual patient profiles, practitioners would further mitigate risks and enhance outcomes [35].

## 6 Enhancing Post‐Laser Care for Microbial Recovery

The recovery phase is a critical window for re‐establishing microbial balance and ensuring long‐term success. Post‐laser care should prioritize products that support the microbiome's natural resilience [36]. For instance, topical probiotics can help recolonize the skin with beneficial bacteria, while prebiotics provide nourishment that encourages their growth [37, 38, 39]. Postbiotics, which are metabolic byproducts of microbial activity, offer additional benefits by directly modulating inflammation and promoting skin barrier repair [40, 41]. Equally important is protecting the skin barrier itself. Ceramide‐rich moisturizers and occlusive agents can shield the skin from external aggressors while maintaining hydration levels conducive to microbial recovery [42]. Patients should be advised to avoid harsh cleansers, alcohol‐based toners, and other products that could further disrupt the microbiome during this vulnerable period [43]. While promising, these strategies require further clinical validation to determine their precise role in post‐laser recovery.

## 7 Case Studies and Emerging Evidence

Recent research highlights the potential of lasers to support the skin microbiome when integrated with microbiome‐conscious protocols. Fractional lasers, such as fractional CO_2_ and Erbium:YAG, create microablative zones of injury surrounded by intact tissue, facilitating faster microbial recolonization. Studies like that of Athanasiou et al. have shown that fractional CO_2_ laser treatments, when combined with postbiotic‐enriched moisturizers, help maintain microbial diversity, reduce dysbiosis, and promote barrier repair [19]. De Sica et al. demonstrated that non‐ablative fractional lasers, such as Erbium glass lasers, are particularly advantageous for preserving microbial health induce dermal remodeling without significantly disrupting the epidermal barrier, enabling faster microbial recovery when paired with microbiome‐supportive skincare [18]. Manolis et al. found that the use of probiotic‐enriched serums following CO_2_ laser resurfacing significantly reduced infection risks and accelerated microbial balance restoration [17].

Further research is needed to optimize these synergistic approaches, focusing on randomized controlled trials to validate their efficacy in maintaining microbial balance.

## 8 Personalization Through Advanced Diagnostics

Personalized medicine is the future of esthetic care, and microbiome analysis is a key tool in this evolution. By sequencing a patient's microbiome prior to treatment, clinicians can identify vulnerabilities, such as low microbial diversity or the presence of pathogenic species. These insights can inform decisions about laser parameters, post‐treatment care, and follow‐up schedules, ensuring that interventions are both effective and microbiome friendly. AI‐driven diagnostic platforms are making these analyses more accessible. By integrating microbiome data with other dermatological parameters, these tools can generate comprehensive treatment plans tailored to individual needs [44]. This level of precision enhances patient outcomes and sets a new standard for care in esthetic medicine.

## 9 The Future of Microbiome‐Friendly Esthetic Dermatology

As our understanding of the skin microbiome deepens, its integration into esthetic practices will likely become the norm rather than the exception. Hybrid treatments that combine lasers with microbiome‐enhancing therapies, such as LED photobiomodulation, represent one exciting avenue for innovation [5]. Similarly, advancements in biotechnology may soon yield post‐treatment products that are specifically designed to support microbial recovery, such as bioactive serums containing live bacteria or their beneficial metabolites. The ultimate goal is to move beyond treating the skin in isolation and adopt a holistic perspective that considers its symbiotic relationship with the microbiome. By doing so, practitioners can achieve results that are esthetically refined yet biologically sustainable, fostering long‐term patient satisfaction and trust.

## 10 Patient Profiles and Predictive Outcomes

The effectiveness and safety of laser treatments are significantly influenced by individual patient profiles. Baseline microbiome health is a critical factor in determining how well the skin recovers post‐treatment [2, 45]. Patients with a diverse and balanced microbiome may exhibit faster healing and lower susceptibility to infections or dysbiosis compared to those with a compromised microbiome, such as individuals with a history of atopic dermatitis or long‐term use of topical antibiotics [46]. Pre‐existing conditions like rosacea or acne may also predispose patients to heightened inflammatory responses, necessitating extra care in selecting laser modalities and parameters. Tailoring laser treatments to accommodate these diverse profiles necessitates a comprehensive understanding of patient history, skin type, and underlying conditions, ensuring personalized and effective outcomes [1, 47]. For instance, patients with rosacea may benefit from non‐ablative or fractional laser modalities to minimize inflammation, while those with compromised microbiomes might require extended recovery periods between sessions. Incorporating these considerations ensures that laser treatments are effective while minimizing risks, fostering safer and more personalized dermatological care.

## 11 Future Perspectives: AI in Microbiome‐Conscious Laser Treatments

The integration of artificial intelligence (AI) into microbiome‐conscious laser treatments offers a transformative opportunity to elevate patient safety and therapeutic outcomes while preserving the delicate skin microbiome. By leveraging AI, clinicians can tailor laser protocols with unprecedented precision, optimizing treatment effectiveness and minimizing microbial disruption. AI‐driven tools could analyze patient‐specific data, including microbiome profiles, skin imaging, and environmental factors, to recommend laser modalities and settings most suited to individual needs [48]. For example, AI algorithms could identify patients at higher risk of laser‐induced dysbiosis and adjust energy levels, pulse durations, or cooling parameters to protect microbial diversity. Machine learning models trained on extensive datasets could also predict complications such as infections or post‐inflammatory hyperpigmentation (PIH), enabling proactive adjustments during treatment planning [49].

During laser procedures, real‐time AI‐enabled monitoring systems could dynamically adjust parameters based on the skin's response. These systems would allow for adaptive treatments that maintain efficacy while minimizing unnecessary damage to the skin barrier and its resident microbiota. Post‐treatment, AI‐powered diagnostic platforms and wearable devices could track microbial recovery and skin barrier restoration [50]. These tools could provide personalized recommendations, such as microbiome‐supportive skincare products or optimal follow‐up schedules, ensuring a faster and safer recovery. Moreover, AI‐driven analyses could help clinicians refine combined treatment approaches, such as pairing lasers with photobiomodulation, to achieve esthetic goals with reduced microbiome disruption.

## 12 Navigating Regulatory Challenges in Microbiome and AI‐Driven Dermatology

The evolving regulatory landscape for microbiome‐conscious laser treatments and AI‐driven applications in dermatology presents critical opportunities and challenges. A key consideration lies in the delineation of claims for microbiome‐related products. For instance, the distinction between cosmetic and therapeutic claims often determines the regulatory pathway, as seen in the Cosmetics Regulation in Europe and the FDA Cosmetic Labeling Guidelines in the United States [51, 52]. Recent studies have highlighted the need for standardized clinical trials to substantiate claims regarding the efficacy of probiotics and prebiotics in dermatological care. For example, França et al. emphasized the role of probiotics in barrier repair and inflammation modulation, suggesting the necessity for robust clinical validation before market approval [41]. Similarly, Gueniche et al. demonstrated microbiome‐supportive product efficacy in enhancing post‐laser recovery, underscoring the need for regulatory oversight to ensure product safety and consistency [36].

The integration of AI in dermatology adds another layer of complexity. The Artificial Intelligence Act (AIA) in the European Union proposes a risk‐based framework for AI applications, which could impact the development of AI‐driven diagnostic tools and predictive models (European Commission, 2024) [51]. Studies such as those by Haykal et al. have demonstrated the transformative potential of AI in tailoring laser treatments to individual microbiome profiles, but these technologies must meet transparency and bias mitigation criteria to ensure equitable outcomes across diverse populations [49]. Regulatory concerns also extend to the ethical use of patient data, as highlighted by the World Health Organization (WHO), which advocates for fairness and inclusivity in AI systems [53].

Regional differences in regulatory approaches further complicate the global adoption of microbiome‐conscious and AI‐driven technologies. While regions like Asia‐Pacific adopt flexible frameworks to encourage innovation, such as Japan's streamlined approval processes for functional cosmetics, disparities in safety and efficacy standards remain (Japan's Ministry of Health, Labour and Welfare, 2024) [54]. The push for global harmonization aligns with sustainability goals, as the UN emphasizes eco‐conscious practices in dermatology, including energy‐efficient devices (UN, 2024) [55]. Addressing these regulatory, ethical, and environmental challenges requires interdisciplinary collaboration and standardized guidelines to ensure innovation and patient safety.

## 13 Conclusions

The interplay between lasers and the skin microbiome represents a burgeoning area of research with immense potential to transform esthetic medicine. Laser technologies have become indispensable tools in modern dermatology, offering highly effective treatments for a variety of skin concerns such as acne scars, hyperpigmentation, and skin laxity. However, the potential impact of these treatments on the skin microbiome, a critical component of skin health, remains underexplored. Ablative lasers, while effective, disrupt the epidermal barrier and microbial equilibrium, while non‐ablative and fractional lasers, though less invasive, still pose risks of microbial perturbation. Adopting microbiome‐conscious approaches is essential to elevate the standard of care. Personalized protocols, informed by emerging insights into the microbiome and supported by advanced diagnostics, could minimize treatment‐associated risks such as dysbiosis, infections, and inflammatory responses. Innovations like combination therapies, including photobiomodulation with fractional lasers, present promising pathways to achieve optimal esthetic results while preserving microbial health. Equally important is the role of post‐treatment care, where microbiome‐supportive regimens featuring probiotics, prebiotics, and barrier‐protective products could facilitate faster recovery and reduce complications.

The integration of AI further amplifies the potential for precision medicine in this field. AI‐driven tools could analyze microbiome profiles, predict treatment outcomes, and enable real‐time parameter adjustments, paving the way for highly tailored and safe interventions. By harmonizing cutting‐edge laser technologies with microbiome science, clinicians can achieve results that are both esthetically refined and biologically sustainable. As research into the skin microbiome and its relationship with lasers advances, the esthetic dermatology community must prioritize the development of evidence‐based protocols that bridge the gap between therapeutic efficacy and microbial preservation. This paradigm shift not only enhances patient safety and satisfaction, but also underscores a broader commitment to holistic and integrative dermatological care. Ultimately, embracing microbiome‐conscious strategies alongside innovations like AI will redefine the future of laser treatments, fostering long‐term skin health while achieving exceptional esthetic outcomes. Future research should focus on clarifying these interactions and developing evidence‐based guidelines for microbiome‐conscious esthetic procedures.

## Conflicts of Interest

The authors declare no conflicts of interest.

## Data Availability

The data that supports the findings of this study are available in the Supporting Information of this article.
